# Editorial: Relationship of language and music, ten years after: Neural organization, cross-domain transfer and evolutionary origins

**DOI:** 10.3389/fpsyg.2022.990857

**Published:** 2022-07-28

**Authors:** Chao-Yang Lee, Caicai Zhang, William Shi-Yuan Wang, Mary Miu Yee Waye

**Affiliations:** ^1^Division of Communication Sciences and Disorders, Ohio University, Athens, OH, United States; ^2^Department of Chinese and Bilingual Studies, The Hong Kong Polytechnic University, Kowloon, Hong Kong SAR, China; ^3^The Nethersole School of Nursing, The Chinese University of Hong Kong, Shatin, Hong Kong SAR, China

**Keywords:** language, music, speech, speech perception, music perception

Language and music are both evolutionarily old and ubiquitous in human cultures. They also share many common features such as the use of basic acoustic attributes, the presence of complex hierarchical structures, and the ability to elicit and communicate emotions. These parallels have sparked questions regarding the neural organization of language and music, the cross-domain transfer between them, and their evolutionary origins. Ten years after the publication of a similar Research Topic in Frontiers, many intriguing questions remain. The 11 articles in this collection address the relationship between language and music from a wide range of perspectives, including six empirical studies on cross-domain transfer, three articles on clinical applications, and two articles on evolutionary perspectives (Figure 1).

**Figure 1:**
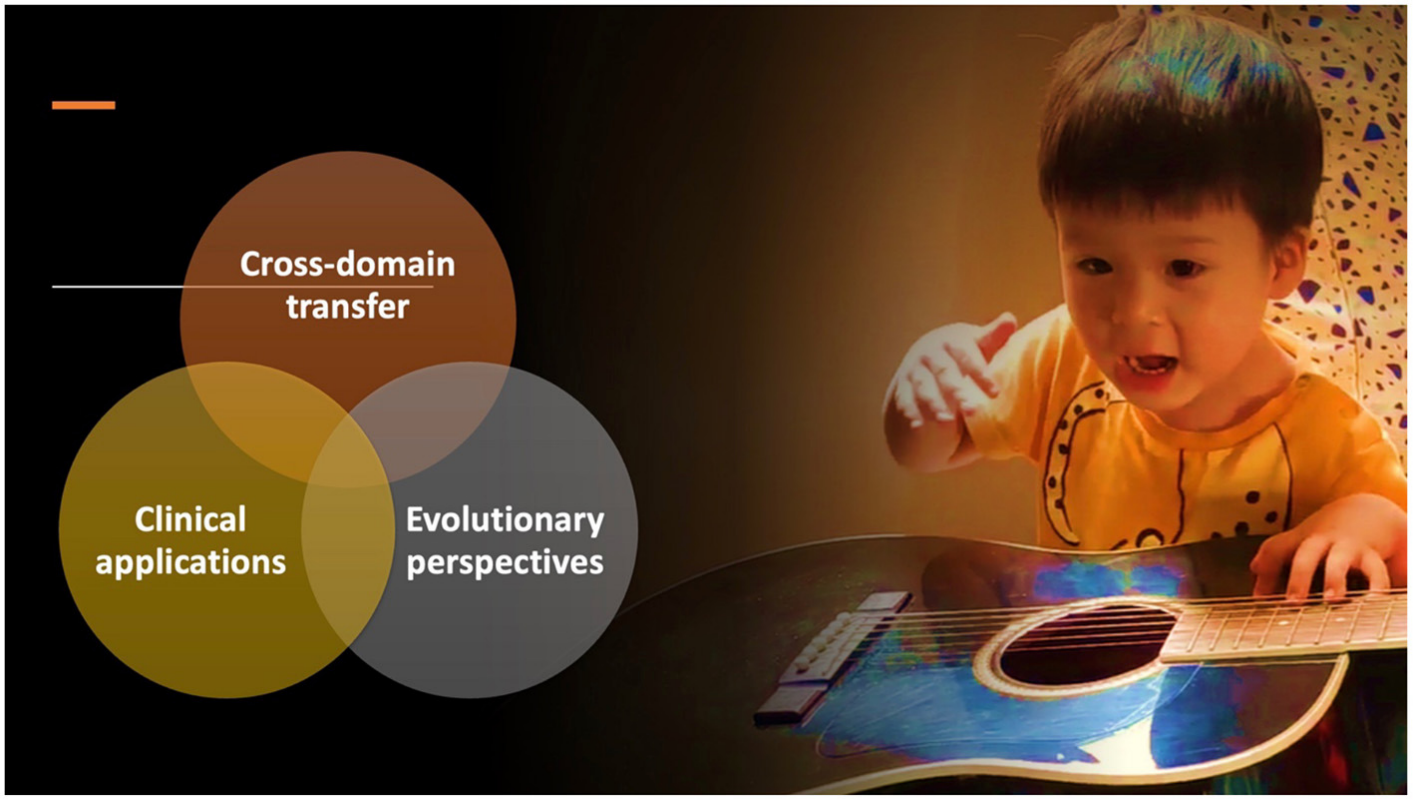
Three areas of study covered by the current Research Topic.

## Cross-domain transfer

A plethora of studies have demonstrated that long-term experience with either language or music can transfer to processing or learning in the other domain. However, the extent and direction of the transfer remains controversial. Several hypotheses have been proposed to explain the transfer, including the domain-general sharpening of sensory encoding (Chandrasekaran and Kraus, 2010), common acoustic processing and influence on abstract representations in the other domain (Besson et al., 2011), and the OPERA/expanded OPERA hypothesis (Patel, 2011, 2014). The use of lexically distinctive pitch in tonal languages has also provided a unique opportunity to evaluate the language-music relationship.

Zhu et al. showed that musicianship affects categorical perception of Mandarin tones by native listeners. Mismatch negativity (MMN) amplitude in response to tonal deviations was amplified in amateur musicians, indicating that musical training enhances pre-attentive processing of lexical tones. This finding also suggests that amateur musical training is sufficient to induce neural plasticity in speakers who already have long-term tonal experience. Chen et al. further showed that musicianship affects categorical perception of Mandarin tones in speakers of a second tone language. Perception of Mandarin pitch direction was more categorical in Cantonese-speaking musicians than non-musicians. The musicians were also more sensitive to stimulus duration and intrinsic F0 associated with vowel quality, suggesting that musicians are able to use their sensitivity to acoustics to form more robust representations of tones in a second language.

The impact of musical experience extends beyond phonetic categorization. Smit et al. found an association between pitch discrimination ability and learning of novel words. Surprisingly, better pitch discrimination was associated with worse word learning. The use of infant-directed speech in this study may have led to greater pitch variation, which turned out to be detrimental to learning for individuals with better pitch discrimination abilities. Nie et al. showed that musical training affects working memory. The authors compared a group of Mandarin-speaking children who received 1-year music training to another two groups of children that received either second-language training or no training. After controlling for initial group differences in the baseline performance, the authors found superior development of auditory working memory in the music group.

Choi examined the other direction of the cross-domain transfer, i.e., how language experience affects musical pitch perception. Cantonese language experience facilitated musical pitch perception, but the effect was limited to non-musicians. The lack of a language effect among musicians was attributed to perceptual saturation due to musical training or specialized pitch processing. With these findings, Choi proposed to revise the “Precision” criterion in the OPERA/expanded OPERA hypothesis to accommodate the language-to-music transfer.

The cross-domain transfer, however, does not appear to apply all the time. Tao et al. used the language-music connection to address a long-standing issue in speech perception: Does speech normalization require a general auditory mechanism or a speech-specific perceptual mechanism? The authors showed that familiarity with a music context did not give musicians an advantage in Cantonese tone normalization. Rather, tone normalization occurred only in the speech context, and there was no difference in tone normalization performance between musicians and non-musicians regardless of the context, suggesting that a speech-specific mechanism is responsible for perceptual normalization.

## Clinical applications

Three articles addressed the clinical application of the language-music connection. Zhang et al. compared the effectiveness of melodic intonation therapy (MIT) and traditional speech therapy on Mandarin-speaking individuals with non-fluent aphasia. After 8 weeks of therapy, patients receiving MIT showed better listening comprehension, repetition, and spontaneous naming compared to those receiving traditional speech therapy. The authors concluded that MIT is an effective approach to rehabilitating language functions. Zhang, Li, et al. offered a systematic review of 39 randomized controlled trials examining the effect of MIT on the treatment of non-fluent aphasia. The review showed that behavioral measurements were used in most of the studies, and few studies provided brain imaging data. With this observation, the authors call for more clinical studies incorporating both behavioral and neurophysiological data to evaluate the effectiveness of MIT.

Zhang, Song, et al. compared the effects of vocal intonation therapy (VIT) to standard respiratory therapy on voice production in people with respiratory dysfunction resulting from cervical spinal cord injury (CSCI). After 12 weeks of treatment, patients in the VIT group outperformed those in the standard respiratory therapy group in measures of speech volume, singing volume, sustained note length, and fundamental frequency, suggesting that VIT is an effective treatment for respiratory dysfunctions in CSCI patients.

## Evolutionary perspectives

Commonalities between speech and music in basic acoustic attributes and hierarchical structure have inspired many researchers to hypothesize a common evolutionary precursor. Previous studies have probed the co-evolution hypothesis from the perspective of pitch (Thompson et al., 2012; Fenk-Oczlon, 2017). Fenk-Oczlon extended the investigation to duration. By showing iconic association between vowel height and the duration of musical notes in 20 Alpine traditional yodels, Fenk-Oczlon provided new evidence for the “musical protolanguage” hypothesis (Darwin, 1871; Fitch, 2005, 2006). The close coupling of vowel height and music in singing with meaningless syllables is perhaps reminiscent of an ancient, prosodic protolanguage.

The evolutionary perspective is elaborated in Haiduk and Fitch. Following Darwin's speculation about the speech-music relationship (Darwin, 1871) and Hockett's “design features” of communication (Hockett, 1960), Haiduk and Fitch delineate the evolutionary circumstances which led to the distinctive trajectories that language and music took in their respective developments. They consider the differences and similarities in evolution when language and music are used in a variety of contexts. These ideas are new probes and need to be nourished with much more interaction and perhaps experimentation across diverse cultures. Cultural evolution works much faster than biological evolution, and humans have become a very different kind of animal since Darwin's time. We are living in a very different physical and social environment of our own making. The trajectories that language and music will take in the decades ahead are hard to predict and bound to be fascinating.

## Ethics statement

Written informed consent was obtained from the individual(s), and minor(s)' legal guardian/next of kin, for the publication of any potentially identifiable images or data included in this article.

## Author contributions

C-YL wrote the first draft of the manuscript. All authors contributed to manuscript revision, read, and approved the submitted version.

## Conflict of interest

The authors declare that the research was conducted in the absence of any commercial or financial relationships that could be construed as a potential conflict of interest.

## Publisher's note

All claims expressed in this article are solely those of the authors and do not necessarily represent those of their affiliated organizations, or those of the publisher, the editors and the reviewers. Any product that may be evaluated in this article, or claim that may be made by its manufacturer, is not guaranteed or endorsed by the publisher.
